# Investigation of the Impact from IL-2, IL-7, and IL-15 on the Growth and Signaling of Activated CD4^+^ T Cells

**DOI:** 10.3390/ijms21217814

**Published:** 2020-10-22

**Authors:** Canaan Coppola, Brooks Hopkins, Steven Huhn, Zhimei Du, Zuyi Huang, William J. Kelly

**Affiliations:** 1 Department of Chemical Engineering, Villanova University, Villanova, PA 19085, USA; ccoppol2@villanova.edu (C.C.); bhopki01@villanova.edu (B.H.); 2 Cell/Gene Therapy and Biologics Development, Merck & Co., Kenilworth, NJ 07033, USA; steven.huhn@merck.com (S.H.); zhimei.du@merck.com (Z.D.)

**Keywords:** RNA sequencing, activated CD4^+^ T cell, interleukin-2, interleukin-7, interleukin-15

## Abstract

While CAR-T therapy is a growing and promising area of cancer research, it is limited by high cost and the difficulty of consistently culturing T-cells to therapeutically relevant concentrations ex-vivo. Cytokines IL-2, IL-7 and IL-15 have been found to stimulate the growth of T cells, however, the optimized combination of these three cytokines for T cell proliferation is unknown. In this study, we designed an integrated experimental and modeling approach to optimize cytokine supplementation for rapid expansion in clinical applications. We assessed the growth data for statistical improvements over no cytokine supplementation and used a systems biology approach to identify genes with the highest magnitude of expression change from control at several time points. Further, we developed a predictive mathematical model to project the growth rate for various cytokine combinations, and investigate genes and reactions regulated by cytokines in activated CD4^+^ T cells. The most favorable conditions from the T cell growth study and from the predictive model align to include the full range of IL-2 and IL-7 studied, and at lower levels of IL-15 (6 ng/mL or 36 ng/mL). The highest growth rates were observed where either IL-2 or IL-7 was at the highest concentration tested (15 ng/mL for IL-2 and 80 ng/mL for IL-7) while the other was at the lowest (1 ng/mL for IL-2 and 6 ng/mL for IL-7), or where both IL-2 and IL-7 concentrations are moderate-corresponding to condition keys 200, 020, and 110 respectively. This suggests a synergistic interaction of IL-2 and IL-7 with regards to promoting optimal proliferation and survival of the activated CD4^+^ T cells. Transcriptomic data analysis identified the genes and transcriptional regulators up/down-regulated by each of the cytokines IL-2, IL-7, and IL-15. It was found that the genes with persistent expressing changes were associated with major pathways involved in cell growth and proliferation. In addition to influencing T cell metabolism, the three cytokines were found to regulate specific genes involved in TCR, JAK/STAT, MAPK, AKT and PI3K-AKT signaling. The developed Fuzzy model that can predict the growth rate of activated CD4^+^ T cells for various combinations of cytokines, along with identified optimal cytokine cocktails and important genes found in transcriptomic data, can pave the way for optimizing activated CD4 T cells by regulating cytokines in the clinical setting.

## 1. Introduction

In immunotherapies, adoptive or engineered T cell therapies are a personalized approach to successful remediation of various diseases. Identifying a robust process that can efficiently induce proliferation of naïve T cells is essential in T cell manufacturing process development. In an article published in 2019 by *Cancer Discovery*, the authors touch on the importance of naïve CD4^+^ T cells in pediatric cancer patients receiving immunotherapies. The effects of repetitive chemotherapy cycles deplete naïve T cells in many pediatric cancer patients, which minimizes the potential to induce proliferation successfully after adoptive cellular therapies [1]. Furthermore, the complete impacts of cytokines–and especially the combinations of cytokines–is poorly understood in the context of the rapid expansion of ex-vivo T-cell cultures and in how specific T-cell subsets differentially respond. As part of the CAR-T cancer therapy, transfected T-cells are grown ex-vivo until therapeutically relevant concentrations are reached. This process can be challenging to do efficiently, as the in-vivo environment normally provides the T-cells with growth-regulating signals that are absent in conventional medium formulations. The addition of the interleukin-2, 7, and 15 cytokines has been shown to induce human T-cell activation proliferation *in-vitro*, although the cytokine concentrations and growth rates are not linearly correlated [2,3]. There is extensive qualitative and less quantitative information on the merits of the cytokines’ impact on T cell proliferation individually per phenotype. There is also lacking evidence in an experimentally design study to reveal the optimal combination and overall impact the cytokines have on the proliferation of the CD4^+^ T cell phenotype [3]. It is useful, therefore, to study the impact of the concentration of the aforementioned cytokines on the proliferation and signal transduction of the naïve CD4^+^ T cells.

Another aim of the study is to generate more data and information for naïve CD4^+^ T cells due to the inherent challenges this phenotype presents. Natural killer (NK) cells and cytolytic CD8^+^ T cells are often more easily influenced with cytokines into proliferation, but the CD4^+^ T cells are less responsive to one cytokine alone [3,4,5,6,7]. According to the literature, there is more to be done to unlock a useful cocktail of cytokines to balance the slow growth of the naïve CD4^+^ T cells and the tendency for this phenotype to undergo differentiation to a different phenotype [8]. CD4^+^ naïve T cells play critical roles in aiding the expansion of adoptive immunotherapies in vivo, and therefore this study can positively impact manufacturing productivity and efficacy while reducing uncertainty of cytokine concentration [9,10].

To develop the conditions for the experiment, the team studied historical reference points that may lead to the appropriate boundary conditions. As naïve CD4^+^ T cells are less responsive than naïve CD8^+^ T cells and NK cells [5], we would need to carefully examine the ranges of application for each of the cytokines. Alves, et al. describes IL-15 being used to stimulate growth of naïve CD4^+^ and naive CD8^+^ T cells, as well as NK cells [5]. In that experiment, naïve CD4^+^ T cells were required at least 40 ng/mL to divide at approximately 20% rate. This led roughly 40 ng/mL to become the median in the condition data set for IL-15.

Geginat et al. note that in particular, naïve CD4^+^ T cells respond quickly to antigen-presenting molecules (APM), having only a 40-h lag prior to first division, when compared with cytokine stimulation alone having a lag of 72 h [3]. The division times are characteristically different in the naive CD4^+^ T cells when exposed to APMs versus cytokines, where the division times averaged 10 h when responding to APMs versus 30 h when responding to cytokines [3]. IL-7 and IL-15 cytokines were used in the study to induce proliferation, whereby the concentration was approximately 25 ng/mL for both cytokines in the study [3]. Another study was able to provide some background for concentrations of IL-2 needed for the study; Litvinova, et al. describes the concentration of IL-2 used to stimulate significant growth in CD4^+^ T cells as 1 ng/mL [11]. When compared to IL-7 and IL-15, IL-2 alone was the only cytokine to drive significant growth. Silva et al., describes the proliferation of naïve CD4^+^ T cell maintenance in patients with thymectomy, using IL-2 and IL-7 to stimulate the growth [12]. The concentration of IL-2 was 2 ng/mL and the IL-7 concentration was 10 ng/mL which successfully drove improved proliferation of the cells. Jaleco, et al, ran an experiment of naïve and memory CD4^+^ T cell subsets, attempting to walk the tight rope of proliferation and apoptosis [4]. The findings show that IL-2 and IL-7 act together for proliferative cell divisions, which is significantly more effective than using IL-2 or IL-7 alone. The concentration of IL-7 was again 10 ng/mL and the concentration of IL-2 was 6 ng/mL. Finally, Dooms and Abbas, as well as Kinter, et al., tested up to 100 ng/mL for IL-2, IL-7 and IL-15, but there were cell death implications of the highest concentration [13,14]. We thus deduce that the optimal concentration lies likely between 1–100 ng/mL for each of the cytokines.

On the basis of the review on the cytokine concentrations used to promote T cell growth, sixteen conditions were designed in this study to investigate the expansion of the CD4^+^ T-cell activated with anti-CD3/CD28 dynabeads under the influence of various combinations of IL-2, IL-7 and IL-15 in a 96-well plate format. Three levels of concentrations, which represent low, moderate and high concentrations of each cytokine, were applied in four experiments. The growth data were thoroughly analyzed with ANOVA tests [15] to identify the cytokine cocktails that were able to significantly enhance the growth of activated CD4^+^ T cells when compared to the control condition. A Fuzzy model, which is known for its good prediction capability [16], was then developed to predict the growth of activated CD4^+^ T cells and study the interaction of cytokines in promoting activated CD4^+^ T cell growth. Finally, transcriptomic data was obtained for each cytokine stimulation and analyzed with principal component analysis [17], GeneMania [18], and MetaCore [19] to identify the genes/reactions regulated by each cytokines in activated CD4^+^ T cell. The genes whose profiles are correlated with the growth rates were also identified. The relevance of this work stems from its innate foundational-level exploration for the advancement of understanding the quantifiable impact of each of the three cytokines on the growth of activated CD4^+^ T cells. Additionally, we sought to study the synergistic impact of different combinations of cytokines on the growth rate of the T cells. To our knowledge, this is the first trial to systematically investigate the combination of the aforementioned cytokines to accelerate the growth of activated CD4^+^ T cells.

## 2. Results

### 2.1. Identify Optimal Cytokine Combinations from the Statistical Analysis of Cell Growth Rates

Cells were thawed from cryopreservation in DMSO and activated with anti-CD3/CD28 dynabeads on day 0 of the experiment, and cytokine supplementation at the experimental conditions was performed on day three after media acclimation and post-activation anergy had completed. Pairwise *t*-tests were then conducted on the growth rates obtained from four datasets for 16 conditions each (Table 1).

**Figure 1:**
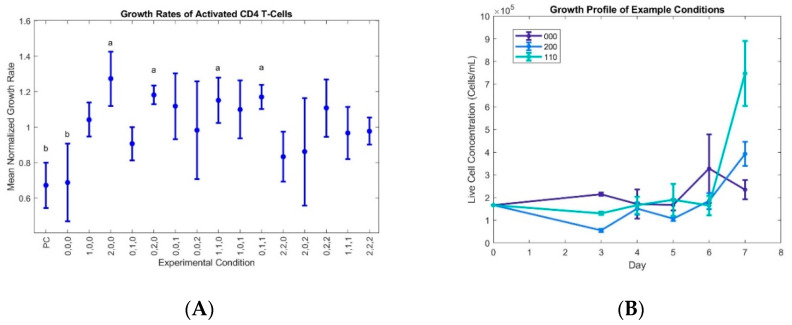
Various combinations of the IL-2, IL-7, and IL-15 cytokines differentially regulate activated CD4^+^ lymphocyte proliferation: (**A**) Mean growth rate data were obtained in four data sets, with growth rates calculated for a four-day period after post-thaw acclimation, and each set normalized to its respective average growth rate. Error bars represent ± 1 SE, and grouping letters show statistically significant differences (α < 0.05) as measured by pairwise *t*-tests. *p*-values are shown in Table 1. Note: “a” in the figure indicates conditions with growth rates significantly higher than the negative control and the baseline 000 conditions; and “b” represents the negative control and 000 conditions (see Table 5 for experimental concentrations). (**B**) Example growth profile of selected conditions, where cytokines were added on day 3 and growths rates calculated between days 3 and 7. Error bars represent ± 1 SD. Full data sets are available upon request.

The growth rates were normalized to the average growth rate for each data set for easy comparison of improved and inhibited growth, and the data show that the growth rates for conditions 200, 020, 110, and 011 are statistically high when compared to the negative control (no cytokine supplementation) and baseline 000 condition (Figure 1).

Since the initially naïve cells will produce endogenous cytokines, the no cytokine control was used as a baseline for the effects of interleukin supplementation. The similarity of the 000 condition to the no cytokine control suggests a critical level of supplementation for initial proliferation that was not met by the low levels of the 000 condition. The condition keys represent the concentrations of each cytokine (IL-2, IL-7, IL-15) at the three different experimental levels, with 0, 1, and 2 in each position indicating the low, medium, and high concentrations, respectively (Table 2).

### 2.2. Investigate the Interaction of the Three Cytokines in Promoting the Growth of CD4 + Naïve T Cells

Factorial analysis of variance on the combined data showed a significant interaction between all three cytokines and stronger interaction of IL-2 and IL-7 specifically (Table 3). All three of these cytokines signal through the common γ chain and have been shown independently to differentially regulate the proliferation of T cell subsets. The interaction of IL-2 and IL-7 is also present and significant in three of the four data sets alone, supporting previous findings of the importance and synergy of these cytokines for the proliferation and survival of naïve CD4^+^ lymphocytes. Co-stimulation with IL-2 and IL-7 has been implicated in modulating the proliferative, apoptotic, and necrotic susceptibilities of naïve T cells [4]. IL-7 alone can promote the survival of naïve CD4^+^ T cells ex vivo, while the combination also increases their sensitivity to Fas-mediated cell death. We hypothesize that higher concentrations of these cytokines may allow the cell death mechanism to begin to dominate, while moderate concentrations allow for faster proliferation after Ag activation without triggering extensive cell death.

IL-15 has also been implicated in the proliferation and survival of T-cell populations, notably through its interaction with the IL-2 receptor [18]. Our growth study suggests that moderate IL-15 concentrations can improve the seven-day growth rate from thaw over similar conditions with high IL-15 concentrations; this may be due to competitive binding of IL-15 with IL-2 through their common IL-2 receptor, with high IL-15 concentration reducing the proliferative impact of IL-2. These three cytokines are regulators of the relative amounts of T-cell subsets in a mixed T-cell population [4], and therefore are capable of sensitizing cells to death-inducing pathways, or to proliferative pathways. In our study of Ag-activated CD4^+^ cells, over-stimulation by IL-15 tended to reduce growth rates, while the cells were more tolerant to any level of IL-2 and IL-7 investigated in this study. High IL-2 and low IL-7 and IL-15 (condition 200) gave the most rapid expansion at the cost of overall viability, while the 011 and 110 conditions nearly as rapidly and had higher viabilities (viability data not shown).

### 2.3. Develop a Fuzzy Model to Predict the Growth Rates of Activated CD4^+^ T Cells for Various Cytokine Combinations

**Figure 2:**
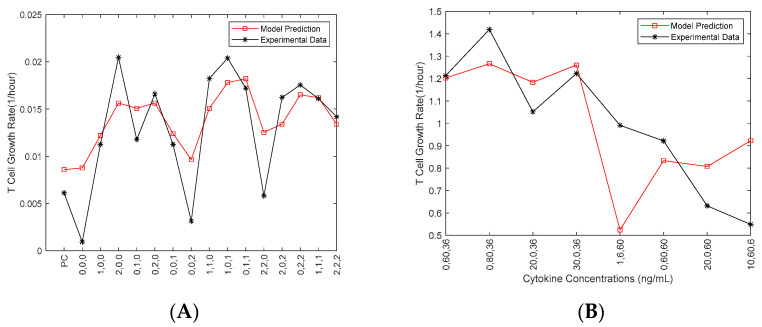
Fuzzy model prediction with experimental data from September (**A**); Fuzzy Model prediction with experimental data from October (**B**). Experimental data in (**A**) is from a single dataset used to estimate parameters for the fuzzy model, and model predictions in (**B**) were tested in a second experimental run to validate the model predictions and analyze the choice of cytokine concentrations for future experiments. MATLAB was used for these modeling studies.

One experimental dataset (obtained in September 2019) was used to estimate the parameters in the Fuzzy model (Figure 2A). The cytokine concentrations from another experimental dataset (obtained in October 2019) for eight new conditions were fed as the inputs of the developed Fuzzy model to predict the growth rate (Figure 2B).

Those eight new conditions, which were not included in the 16 designed conditions, were selected from the fuzzy model to evaluate the model for the conditions returning high, moderate and low growth rates, respectively. Amid the inconsistencies in the above figure can be seen, the model was tested against a different set of conditions that had different levels of cytokines compared to the original data sets. The objective was to validate the spectrum of levels that could be seen in a singular test of the integrity of the 3-D scatter heat map. The model does show some consistency with the data in this case. There is truly only one data point that is inconsistent from the experimental data. The point of cytokine levels 1, 6, 60 ng/mL for IL-2, IL-7 and IL-15, respectively, most closely resembles the original condition 0,0,2, which in data set I was one of the lowest performing conditions (as seen in Figure 2A). The data shown in Figure 2B (i.e., the October 2019 data) was not included in Figure 1. Only one of the other three datasets (i.e., September 2019 data) was used in Fuzzy modeling (Figure 2A), as we aimed to develop the model to validate our choice of the cytokine concentrations and investigate the interaction of the three cytokines before more experiments were conducted.

**Figure 3:**
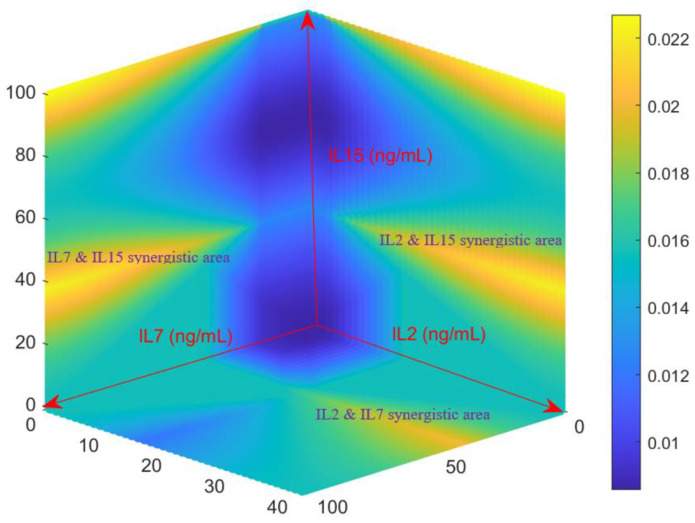
The growth rates of activated CD4^+^ T-cells (hr^−1^) predicted by the model for different combinations of cytokines. The model was assessed over the entire concentration space investigated by the growth studies to generate this map, with all possible combinations of IL-2 ∈  [0–40 ng/mL], IL-7 ∈ [0–100 ng/mL], and IL-15 ∈ [0–100 ng/mL]. The growth rate scale in (hr^−1^) are shown on the right, with yellow representing fastest proliferation and dark blue the lowest.

Since the developed Fuzzy model has been validated by experimental data, it is thus of value to predict the growth rates of activated CD4^+^ T cell growth rates for various combination of IL-2, IL-7 and IL-15. A 3-D surface heat map was generated in Figure 3 from the Fuzzy model by evaluating all possible combinations of IL-2 (0–40 ng/mL), IL-7 (0–100 ng/mL), and IL-15 (0–100 ng/mL). Consistent with what was observed in Section 2.1 and Section 2.2, the Fuzzy model predicts that moderate concentrations of the three cytokines can return acceptable growth rates of activated CD4^+^ T cells (as shown in the middle left and right of the heat map). As implied by the data shown in Figure 2A, the Fuzzy model returns high growth rate for high concentrations of the cocktails IL-7~IL-15 (the left top of Figure 3) and IL-2~IL-15 (the right top of Figure 3). Figure 3 also indicates strong synergistic effect between IL-7 and IL-15 and between IL-2 and IL-15. Additional synergistic effect was also observed for IL-2 and IL-7 in Figure 3. This is also consistent with the result shown in the previous section.

### 2.4. Investigate Genes Indicating the Purtabation in the Metabolism of Activated CD4^+^ T Cells Stimulated by Individual Cytokines

The expression variation of genes that are involved in positive proliferation and negative proliferation were identified in Table 4 using the program Metacore, along with overlapping the up/down-regulated genes with several databases (such as Biological Magnetic Resonance Data Bank and Mammalian Metabolic Enzyme Database). One of the more important aspects we monitored was determining what metabolic pathways were persistently upregulated with high-magnitude fold-increase from baseline genes represented. Using genes from the glycolysis, inositol phosphate, glutamine metabolism, PI3K-AKT, mTOR, Myc, TCA cycle, protein synthesis and glycerophospholipid pathways that are well-known for their important roles in regulating cell metabolism, we analyzed the genes which have ties to these pathways, specifically which are up and down-regulated by each of IL-2, IL-7 and IL-15.

Genes associated with IL-2 stimulation resulting in gene upregulation from the aforementioned metabolic gene pathways include genes associated with the metabolism of lipids, carbohydrates and proteins, including *APOE*, *PFKFB1*, *IGFBP2*, *RPS9* and *PGAM4*. Consequently, one gene downregulated via IL-2 stimulation was *CHST1*, involved in amino acid metabolism. Genes upregulated via IL-7 stimulation include genes involved in carbohydrate, lipid, protein and amino acid metabolism as well: *APOE*, *PFKFB1*, *CES3*, *IGFBP2*, *DMGDH* and *KLK2*. Genes associated with IL-7 stimulation resulting in downregulation include *CHST13*, *CHST1*, *MGIL*, and *ACSM1*. Finally genes associated with IL-15 upregulation are focused in mostly in lipid metabolism: *ACHE*, *APOE*, *RDH5*, *PRKAA2*, *CDS1*, and *DMGDH*. Those genes downregulated by IL-15 include *MRM1*, *ENPP* and *MT5C1A*. Key genes from these lists are compiled in Table 4.

In order to elucidate the functional differences between cytokines and their response to cell growth, we next analyzed significantly changed transcripts associated with proliferation related ontology vs. the control condition (Supplementary Figure S1). All cytokines shared commonalities via induction of HILPDA, a gene which enhances cell growth via lipid metabolism and potent downregulation of SFRP1, a gene involved in delay of S phase. Detailed analysis identified a common set of factors closely shared between IL-2 and IL-7. These shared genes revealed upregulation to PGDFB, JAK/STAT (EPOR), and Beta-catenin (PTPRU) related signaling as well as dramatic negative changes to angiogenic factors (VEGFC, FGF18, FGF2). Conversely, IL-17 resulted in a dramatic increase of TGFB2, downregulation of ERK signaling (GAREM2), and resulted in a decrease in expression of transcriptional programmer, CHD5.

### 2.5. Study Genes Indicating the Purtabation in the Signal Transduction of Activated CD4^+^ T Cells Stimulated by Individual Cytokines

The genes with ± 50% change in their expression levels when compared to the control condition were identified via the approaches shown in the Methods section. Since hundreds of genes were up/down-regulated by each of IL-2, IL-7 and IL-15, principal component analysis was implemented to project those genes according to their expression time-profiles onto a two-dimensional space. The outlier genes were analyzed to identify the reactions unique to each cytokine, while the bulk genes were further analyzed by the program Metacore to identify the gene-regulatory networks induced by each of the three cytokines.

#### 2.5.1. Outlier Gene Analysis

**Figure 4:**
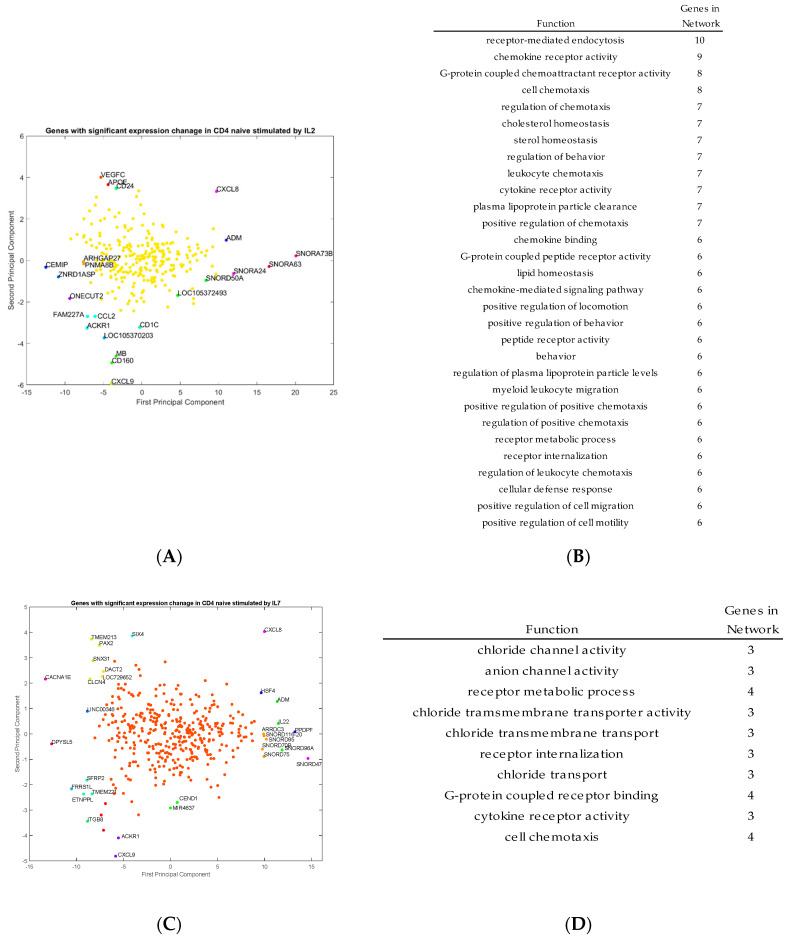

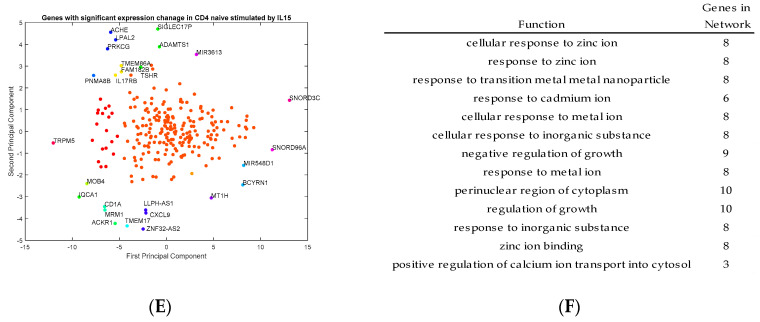
Principal component analysis (PCA) projections of genes with significant expression change by IL-2 (**A**), IL-7(C) and IL-15 (**E**) stimulation, with corresponding and respective gene function of IL-2 (**B**), IL-7 (**D**), and IL-15 (**F**) in activated CD4^+^ T cells. IL2 mainly regulated genes related to cell and leukocyte chemotaxis, chemokine receptor activity, cellular defense response and cell motility, while IL7 regulated genes involved in chloride transport, cell chemotaxis, chloride transmembrane activity, and cytokine receptor activity. The genes regulated by IL15 were involved in cell growth, zinc ion responses, cellular response to metal ions, and regulation of calcium ion transport into the cell.

Outlier genes were identified by principal component analysis and clustering from the transcriptomic data for activated CD4^+^ T cells stimulated by IL-2, IL-7, and IL-15, respectively (Figure 4A,C,E). The functions of those outlier genes were identified from the program GeneMANIA and listed in Figure 4B,D,F. In Figure 4B, the gene functions listed represent unique outlier genes from the PCA study of genes with significant expression change via stimulation by IL-2. The gene functions of the genes stimulated by IL-2 seem to be related to cell and leukocyte chemotaxis, chemokine receptor activity, cellular defense response and cell motility. In Figure 4D, the gene functions listed based on the outlier genes from IL-7 stimulation, which include genes involved in chloride transport, cell chemotaxis, chloride transmembrane activity, and cytokine receptor activity. Finally, in Figure 4F, the gene functions listed based on the outlier genes from IL-15 stimulation include genes with functions, including cell growth, zinc ion responses, cellular response to metal ions, and regulation of calcium ion transport into the cell. These gene functions are consistent with the types of genes which are outliers in the PCA study.

As shown in Figure 4A,B, the genes with respect to IL-2 stimulation include: *CCL2*, *APOE*, *MB*, *CXCL9*, *IL8*, and *ACKR1*. In particular, *CCL2*, *CXCL9* and *CXCL8/IL8* are all markers of proliferating cells. Some of the standout genes include *MB* (myoglobin), as well as *ACKR1*, which is a chemokine receptor. It seems that most genes stimulated by IL-2 appear to be associated with inflammation processes, but also growth pathways and triggers for proliferation. Outlier genes associated with IL-7 include: *CXCL8*, *CXCL9*, *IL-22*, *CACNA1E*, *ADM*, *SFRP2* and *ITGB8*. Although there are some genes in this list which are involved in inflammation and are directing agents, there are also genes associated heavily with ion transport from the cell with some trans-membrane proteins and channel proteins. Outlier genes associated with IL-15 include many metal-ion associated proteins like *MT1H*, and also signaling for other cytokines like *IL17RB*.

While Figure 4 shows the genes regulated by individual cytokines, these outlier genes are commonly involved in T-cell receptor (TCR), a complex of integral membrane proteins on the surface of T cells that takes part in the activation of T-cells in response to an antigen. Genes from pathway analysis through NCI illustrate the complexity of TCR signaling, which leads to proliferation and differentiation of activated CD4^+^ T cells [20]. In particular, the outlier genes shown in Figure 4, especially those regulated by IL-2 and IL-7, were found to be related to related to TCR signaling, as shown in Table 5. It is known that TCR signaling is an important step in activation of activated T cells in order to induce the proliferation and differentiation pathways [20]. The genes being produced in Table 5 as identified in the PCA are relevant as they are connected to the genes associated with direct TCR signaling. For example, CXCL8 is an important chemokine as a directing agent, involved in chemotaxis [21]. VEGFC is a growth factor connected to the MAPK and PI3K-AKT signaling pathways, which can lead to proliferation of T cells [22]. Unfortunately, it is also associated with tumor immune escape in certain cancers [22]. In another article involving VEGFC signaling, blocking VEGFC inhibits infiltration of CD4^+^ T cells, but does not affect T cell cytokine responses in the epithelium of rats burdened with obliterative airway disease (OAD), which could suggest the ability of VEGFC to act as a marker or directing agent for CD4^+^ T cells [23].

In addition to the outlier genes, the bulk genes not specific in Figure 4 are further investigated to derive the gene regulatory networks stimulated by each of the three cytokines.

#### 2.5.2. Bulk Gene Analysis

Next, in order to further illustrate the effects of these cytokines on alternative pathways, we utilized the Metacore program to the bulk genes shown in Figure 4 to calculate the most statistically affected genetic networks either shared (Supplementary Figure S2A) or uniquely changed (Supplementary Figure S2B) between conditions. Common amongst all was a strong angiogenic response, in addition to changes to cell morphology, adhesive properties, as well as EMT remodeling. Alternatively, the differentially expressed networks revealed a clustering of common components to IL-2 and IL-7 stimulation but not IL-15. This included the anti-apoptotic response, JAK/STAT induction, and alteration to chromatin. Conversely, these changes were largely absent from IL-15 signaling.

**Figure 5:**
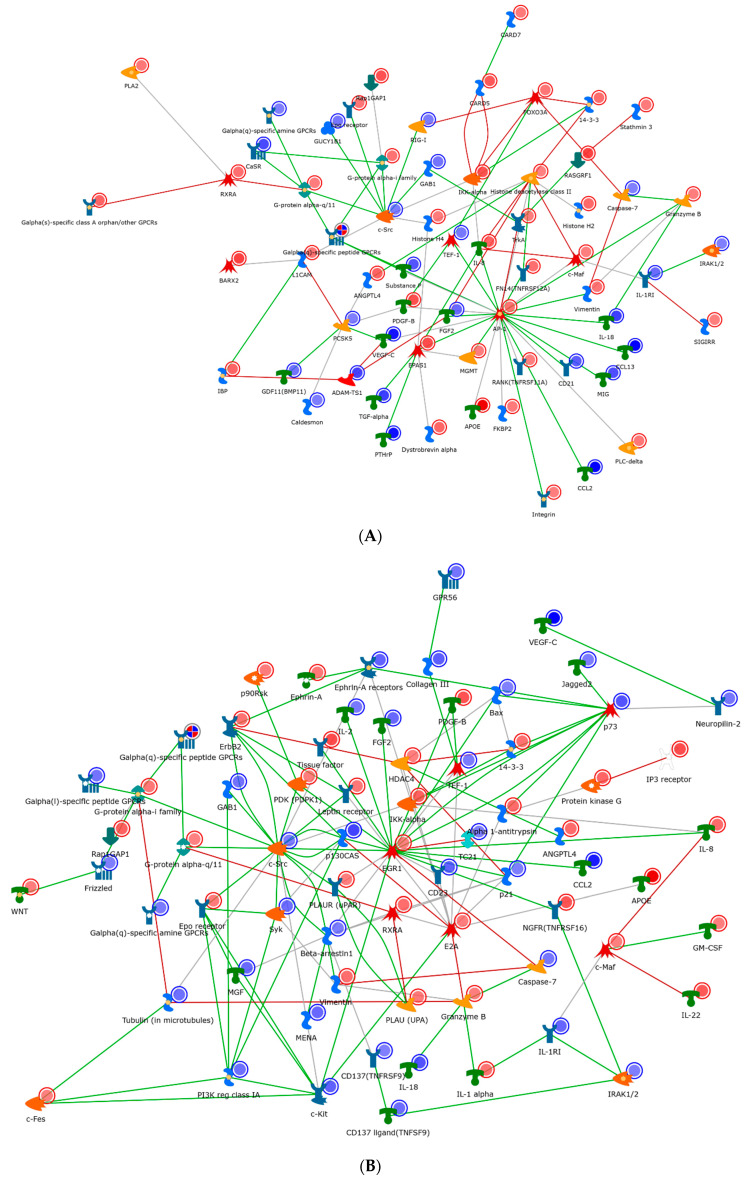

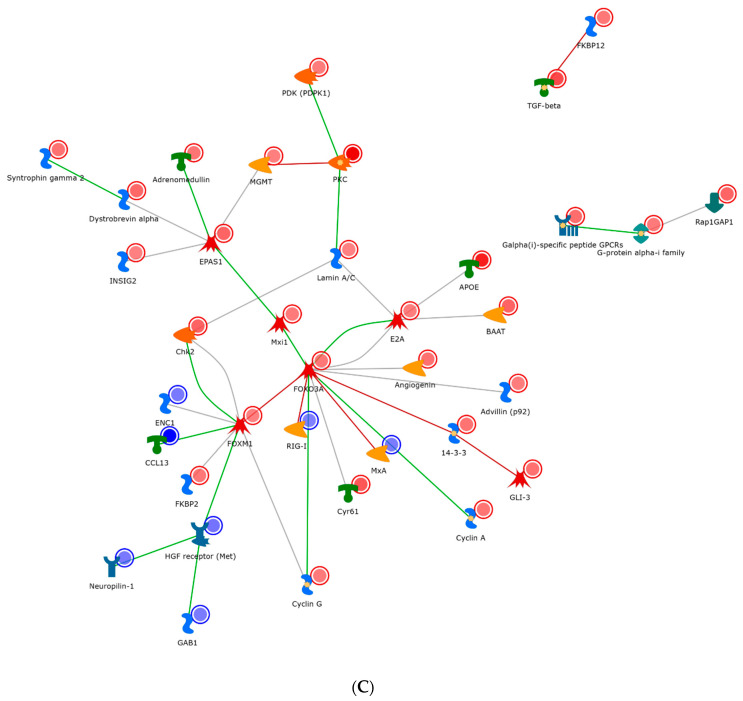
Gene regulatory networks stimulated by IL-2 (**A**), IL-7 (**B**), and IL-15 (**C**) in activated CD4^+^ T cells. The meaning of the icons/modules shown in this figure can be found in the software MetaCore. The IL-2 network was clustered through the oncogene Src, general GPCR signaling, and the transcription factor AP-1. The IL-7 network shared many similar factors as the IL2 one, but it included and expanded repertoire of nodes, specifically around the transcriptional regulators (EGR1, P73, and E2A). The IL-15 network appeared the most dissimilar to IL-2/7 and radiated through a hub of the transcription factors FOXM1 and EPAS1 and cellular morphogenesis.

**Figure 6:**
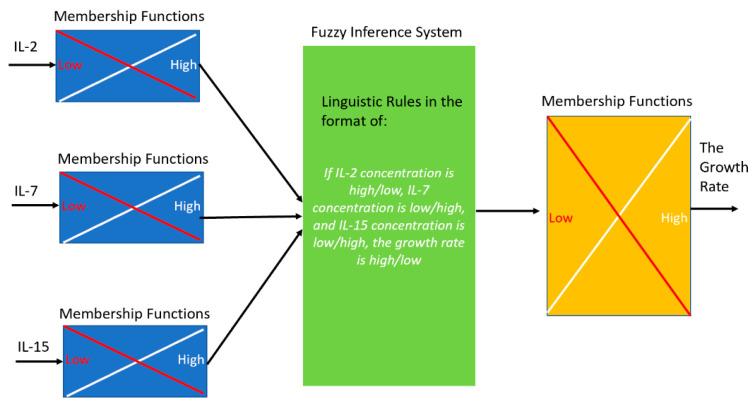
An illustrative diagram of the fuzzy model to link the cytokine concentrations to the T cell growth rate. Membership functions were used to evaluate the possibility of each cytokine to be low or high (i.e., fuzzification), which is followed by the linguistic rules (i.e., inference) to determine the growth rate (i.e., defuzzification).

In order to further describe these networks, we then generated process networks from the differentially expressed genes and visualized only the nodes demonstrating interconnection (Figure 5). The IL-2 network (Figure 5A) demonstrated a network clustered through the oncogene Src, general GPCR signaling, and the transcription factor AP-1. Change in chromatin modelling was noted via a variety of histone deacetylases. Several other transcription factors and molecules which are involved in IL-2 metabolism are also shown in Figure 6A. For example, c-Jun is an example of a transcription factor subunit, marked by the AP-1 transcription factor, which is known to be involved in proliferation, differentiation, apoptosis and survival. HDAC4 and HDAC5 are histone deacetylases, which act as transcriptional repressors. Finally, c-SRC is a tyrosine protein kinase involved in cell-growth.

As compared to IL-2, the IL-7 network (Figure 5B) shared many similar factors, but included and expanded repertoire of nodes, specifically around the transcriptional regulators (EGR1, P73, and E2A). These regulators appeared to have obvious effects within the cell cycle (P21). In particular, EGR1 is a zinc finger protein involved in transcriptional regulation and P73 is a tumor suppressor protein associated with P53. Conversely, the IL-15 network appeared the most dissimilar to IL-2/7 and radiated through a hub of the transcription factors FOXM1 and EPAS1 and cellular morphogenesis (Figure 5C). Specifically, FOXM1 is a transcription factor associated with cell proliferation, and EPAS1 is involved in the induction of genes regulated by oxygen. In addition to FOXM1 and EPAS1, other transcription factors are also found important in Figure 5, such as FOXO3A (a transcription factor associated with apoptosis) and PKC (a transcription factor involved in cell adhesion).

### 2.6. Identify Genes Whose Expression Profiles Were Closely Correlated with the Growth Profiles of Activated CD4^+^ T Cells

The correlation between genes whose growth time profile matched the growth rate delivered for the respective experiment condition were conducted to identify the genes that may be of clinical value for genetic engineering of T cells. The T cell growth data and transcriptomic time profile data were taken for days 4, 5 and 7 to determine whether there were changes to the transcriptome over the course of the experiment and to compare to the growth profiles of activated CD4^+^ T cells. The genes showing the most similar time dynamics as the growth rate are found for each of the three cytokines. Up-regulating these genes may be helpful to promote the growth of activated CD4^+^ T cells. Genes with the most similar dynamics as compared to the growth rate of the cell during IL-2 stimulation were analyzed. The same approach was taken for genes upregulated and matching the profile of growth with IL-7 stimulation, and subsequently the same completed with genes upregulated by IL-15.

Genes whose growth profiles most similarly match the T cell growth when stimulated by IL-2 include genes such as: TMEM74B and TTC36, a transmembrane protein and a tumor suppressor. IL-2 is known to be involved in many of the signaling pathways involved in growth and proliferation. Parallel to growth pathways, apoptosis is closely tied. It is not shocking, therefore that there would be genes involved in tumor suppression. Moving on to IL-7, genes whose growth profiles most similarly match the T cell growth when stimulated by IL-7 included ZNF665 and CA9, which are zinc finger proteins and zinc metalloenzymes, respectively. Zinc finger proteins have numerous functions in the human body, including participating in signal transduction, transcriptional regulation and other key metabolic processes. Additionally, another gene upregulated by IL-7 stimulation include ICAM4, which in involve intercellular adhesion. T cells normally form clusters with cells sticking together upon proliferation. Genes whose growth profiles most nearly matched the T cell growth when stimulated by IL-15 included genes such as PFKFB1, which is involved in glycolysis activation, and TPTE2, which is involved in inositol phospholipid metabolism, which would be used to form cell walls.

The aforementioned genes which are similar to the growth rate of T cell stimulated solely with IL-2, IL-7 or IL-15, represent the polarizing conditions of the T cell, which would be the highest concentrations of IL-2, IL-7 and IL-15, respectively, which were compared to the control condition. This was done to see the numerical fold-increase from baseline for the time profile. Results from this study were also able to help delineate whether the genes were persistently upregulated compared to control, or changed the magnitude of the concentration as compared to control.

## 3. Discussion

### 3.1. The Impact of Each Cytokine on T Cell Growths

IL-2 and IL-7 had the largest impact on growth rates, however high levels of both did not result in the highest growth rates for this study. 15 ng/mL IL-2 with 6 ng/mL IL-7 (condition 200), 1 ng/mL IL-2 with 80 ng/mL IL-7 (condition 020), and 10 ng/mL IL-2 with 36 ng/mL IL-7 (condition 110) provided the best growth rates, and were statistically indistinguishable from each other. IL-15 was constant at 6 ng/mL for each of these top performers. Condition 011, corresponding to 1 ng/mL IL-2 and 36 ng/mL IL-7 and IL-15, was also among this statistical group of highly proliferative conditions.

Much of the literature stipulated that there was a positive correlation between growth rate of CD4^+^ T cells and the presence of IL-2 and IL-7. The condition that would correlate with higher concentrations of these two cytokines is 110, which produces the third highest growth rate given the experimental data in Figure 2A, and the highest growth rate in Figure 1 after normalization. The conditions that produced the highest growth rate were 200 (highest IL-2) and 020 (high IL-7 and low IL-2 and -15) which was surprising. All conditions with IL-2, IL-7, and IL-15 present out-performed the control with none of these present; however moderate levels of each cytokine performed better than the extremes. The 000 condition (lowest non-zero concentrations of each cytokine) did not statistically perform any better than the control, and the 222 condition was very close to the average growth rate. This suggests that an optimal range for each cytokine exists, and over-stimulation by any cytokines may reduce proliferation of *in-vitro* activated CD4^+^ cultures with respect to the initial (seven-day), post-thaw cultures important for the CAR-T process.

### 3.2. The Factors that May Contribute to the Uncertainties in the Growth Rate Data

After a third dataset was collected, we pooled the data for each of the conditions to demonstrate average findings over time between the data sets, presented in Figure 1. The pooled data set does illustrate some uncertainties between the first two data sets and the average of all four data sets. There are a few possible reasons that this could be so, including: differentiation, donor specificity, T cell quantitation method, and nutrient exhaustion. Additionally, the cells were not co-activated with IL-2 until day 3 of the experiment, after a period of acclimation resulting from cryopreservation. The cells, although all from the same donor, may not have responded the same to cryopreservation, lending some uncertainty between data sets. Normalizing the data to the average growth rate of that set improved reliability of condition-to-condition comparisons.

The first potential reason for the growth rate uncertainties is the possibility of cell differentiation. Activated T cells are undifferentiated to a certain extent, not yet at their final functional stage. It is known that cellular signaling pathways which are involved in proliferation are also intertwined with those involved in differentiation [2,8,24]. Another factor for the uncertainties shown in growth data is donor specificity. Donor specificity is always of interest in this type of experiment, because the consistency of T cells from an individual is a controlling factor in the consistency of the results. Patient to patient, there can be unexplained differences in how T cells react depending on gene expression in those specific patients. The data presented here represents two distinct donors, and initial viability from thaw was variable among experimental runs. It is a possibility that the cells could have been less responsive to the stimuli than in the previous run since the data points fall within a narrow standard deviation of each other.

Another reason for the possible discrepancy between the data sets is the possibility of limitations of the method of quantitation, being that it was generated using hand-count techniques in hemocytometer. Using a hand-counting method would introduce potential variance and inconsistency in the measurement system via reproducibility, but also in repeatability when up against an analytical instrument. We did not conduct a gage R&R on the measurement system to confirm the assumption, but moving forward, verifying the accuracy and precision using a gage R&R would likely help remove human error as a factor in considering data uncertainty. Though, the TC20 Automated Cell Counter from Bio-Rad was unable to distinguish CD3/CD28 stimulation beads from activated CD4 T-cells, and gave unreliable results in the absence of stimulation beads due to the small diameter of recovering activated cells. This is why a hand-counting method was implemented in this work.

### 3.3. Discuss Major Findings in Transcriptomic Analysis (What Genes May Be of Clinical Value)

There are some major findings in the transcriptomic data analysis. The first major finding was that many of the genes with persistent genes expressing changes in magnitude above control were associated with major pathways involved in cell growth and proliferation. This finding alone is helpful because the genes are markers that indicate how the cytokines impact the signaling within the cells. Metabolic pathways including: glycolysis and carbohydrate metabolism, lipid metabolism, mTOR, TCA cycle and PI3K-AKT are all pathways with which the up-and down-regulated genes were found to be associated, those of which are important for cell growth [22].

Genes specifically having to do with glycolysis would be a target of clinicians because glycolysis is necessary to fuel the cell during proliferation. Anabolism and cell division are costly processes to the cell, requiring a great deal of energy. With fuel for growth from glycolysis, PFKFB1 is an enzyme regulating the synthesis and degradation of fructose-2,6-bisphosphate. This gene was persistently upregulated in response to IL-15. Another gene of interest with clinical merit would be *CXCL8*, or interleukin-8 (IL-8). IL-8 is an inflammatory chemokine with neutrophil specificity, which can be seen as a chemo-attractant molecule [25]. From the outlier genes found from IL-2, IL-7 and IL-15, this outlier gene is common to both IL-2 and IL-7 stimulation in the transcriptomic data, and has association with many genes involved in TCR signaling. Another closely-related chemokine, CXCL9 was found to be upregulated by IL-2, IL-7 and IL-15. This molecule is of particular interest due to its involvement in immune cell migration, differentiation and activation [25]. Literature suggests that CXCL9 may be a target for cancer therapy as it is a tumor suppressor, and, coupled with other chemokines, can act as a potent differentiator to T helper cells in naïve T cells [26]. VEGFC, or vascular endothelial growth factor-C is another target gene of importance. It was upregulated upon IL-2 stimulation. The literature suggests that this compound has the ability to upregulate MAPK and AKT signaling pathways, both of which are direct links to cellular proliferation. Additionally, the VEGFC pathway is a pathway in itself, which is involved in directing growth of the lymphatic endothelial cells for transport throughout the body [27]. *CCL2* was an outlier gene of the stimulation by IL-2. This gene is involved in T cell differentiation and is a monocyte chemo-attracting protein [28]. It could be a target of clinical value to preclude in the future for preventing differentiation.

### 3.4. The Complexity and Uncertainty of Naïve T Cell Growth, Activation and Pre-Differentiation Phenotyping

This study focuses exclusively on the naïve CD4^+^ T cell phenotype. The cells were thawed and permitted to acclimate to the new post-thaw environment prior to activation and cytokine supplementation. Activation, however, does not consign differentiation, so although the naïve T cells were activated, they do not have to be referred to a specific T-cell subset. In a review published in *Frontiers*, the authors clearly state that, “after development in the thymus, conventional T cells exist as naïve, or undifferentiated cells, and upon activation can undergo clonal expansion and wield different effector functions,” [29]. Effector functions can serve to be a multitude of directions from a cellular cascade perspective. There are T-reg cells, Helper T Cells, Follicular Helper cells and an effector function can be a capability of cytotoxicity. Further, the authors state, “a trivial quantity of activated cells become memory cells,” which again implies that differentiation is downstream of activation [29]. Additionally, activation by cytokines and other chemokines differs drastically as compared to a specific antigen or pathogenic material. The most drastic difference noted by the authors in *Frontiers* is in the modulation of the survival programming, surrounding pathways such as apoptosis and necrosis [29]. Naïve T cells and their activated counterparts differ in this arena but it does not consign a different phenotype than the naïve phenotype initially. This is the main justification for using “activated” T cells, instead of “naïve” T cells throughout the paper.

### 3.5. Design of Experiments

The design of the experiments included, with the exception of the negative control, conditions including at least a low level of each cytokine in the mix, without single cytokine conditions. The four key reasons why single-cytokine groups were not implemented in this experiment include: first, IL-2, IL-7 and IL-15 all share the γ-chain in CD4^+^ lymphocytes, but the threshold for stimulation clearly is above the 000 level for all cytokines presented by the data and supported by literature. Additionally, it is also clear that a tertiary cocktail of all three γ-chain cytokines does not work in a synergistic way to raise the growth rate, as is evidenced by the “222” condition. Second, singular cytokines tend to have significant impacts by themselves with particular subsets of CD4^+^ T cells, i.e., IL-7 is pertinent for survival of memory T cell subsets, but not exclusively key for CD4^+^ naïve T cell phenotype expansion. The saturation of IL-7 in memory T cell survival is evidenced in the literature. Third, certain cytokines, such as IL-2 are known at higher concentrations to induce apoptosis, yet when coupled with other cytokines, there tends to be a synergistic effect [4]. Fourth and finally, the last reason we use combinations of cytokines is to reduce the concentrations of certain cytokines relative to one another. The impact of single cytokines can place cells down specific signaling pathways, which in reality is both unlikely to take place in vivo during lymphocyte expansion, and also is not likely to happen under normal cell conditions in humans. Many cytokines are present at once, all with their own individual concentrations; some are naturally occurring below 1ng/mL and others are present above 1ng/mL depending on the impact the cytokine has on a particular cell type. The cytokines we discuss here are pleiotropic in nature, so their impact specifically on CD4^+^ T cells is different than on other cell types.

### 3.6. Cytokine Storm, Cytokine Release Syndrome (CRS), and Relation to Immunotherapies, SARS-CoV-2

During the discovery phase of the testing of chimeric antigen receptor (CAR) T cells, one of the major findings was the consistent violent reaction of a patient’s own immune system to the engineered T cells [30]. History has shown that a patient’s own immune system can turn against the host when activated incorrectly by a pathogen or antigen-presenting cell (APC), such as during the 1918 Spanish Pandemic Flu. The severity of this pandemic was a result of the immune system’s unregulated cytokine release, which is termed a “cytokine storm.” Scientists later observed this phenomenon again during a disastrous failed Phase I clinical trial of CD-28 monocloncal antibody (mAb) superagonist TGN1412, where all six volunteers were plagued with cytokine storm shortly (minutes) after administration of the novel mAb [31]. Scientists have a better understanding of CRS and cytokine storm associated with especially the T cell immunotherapies now that there is a diverse set of data relating to different types of cancers as well as different demographics [32]. CD-19 blood-borne cancers like leukemia variants seem to be the best responders to CAR-T immunotherapies, especially in pediatric patients, who’s adaptive immune systems are underdeveloped and less coordinated [30]. Also, cancer load seems to play a large part in the severity of CRS for T cell immunotherapy patients’ immune systems to react proportionately [30]. Those with lower disease load or “burden” are less susceptible to CRS [31]. Although therapeutic drugs like Tocilizumab (IL-6 inhibitor) and Daclizumab (IL-2 inhibitor) have been shown to reduce CRS grade [31,32], new studies have shown, specifically that anti-inflammatory cytokines like IL-37 and IL-38 have inhibitive effects on IL-1, which in the recent SARS CoV-2 (COVID-19) pandemic outbreak has played a part in our understanding of cytokine release and cytokine-related inflammation [33]. CAR-T therapies also have an inductive effect on IL-1, the pro-inflammatory cytokine, which as previously mentioned, can potentially be neutralized using the anti-inflammatory effect of IL-37 [34]. Finally, one of the root sources of the IL-1 induction by SARS-CoV-2 seems to be mast cell activation by the virus itself [35]. The mast cell is a histamine-producing part of the innate immune system, rather than the adaptive immune system with T cells, however, recognition of pathogens by these mast cells ultimately impacts the adaptive immune system through cytokine signaling. Using anti-inflammatory cytokine signaling, there is a possibility to reduce the high levels of inflammation seen in CAR-T immunotherapies.

### 3.7. Limitation of This Work

There are a few limiting factors of this work. Given there was a shortage of cells for the experiments, triplicates of each of the 16 experimental conditions were not available. The shortage of cells created uncertainty in the data sets both from a replicability, and also reproducibility perspective. Additionally, and perhaps of equal relevance, the RNA sequencing data that was able to be gathered was not complete due to the limited time and available resources, in the sense that only the effects of individual cytokines were able to be analyzed. There were no coupling or interactive effects that could be studied. This has particular relevance in that the conditions identified as most proliferative in the study were not able to be evaluated because cytokine coupling was not part of the testing completed.

Suggestions for future work would be several recommendations. The first recommendation would be to take measures to minimize the uncertainty in the data sets. The uncertainty was explained, however maintaining the same donor throughout the experiment sets would be one suggestion to reduce variance. In addition, verifying the measurement system from the perspective of replicability versus reproducibility is especially important in developing consistent results. Other T-cell phenotypes will also be investigated, and preliminary data has been collected for CD8 subsets, as they tend to be favored for CAR-T processes currently. Finally, RNA Sequencing should be conducted for more experimental conditions, especially those conditions with various cytokine combinations.

## 4. Materials and Methods

### 4.1. Cell Culture and Cytokine Stimulation

1-mL vials of naïve CD4^+^ T-cells from a human donor were purchased (Astarte Biologics, Bothell, WA, USA) and stored in the vapor phase of liquid nitrogen until ready for culture. The cells were thawed in a 37 °C water bath for 1–2 min or until pipettable, transferred to a 15-mL conical tube, and resuspended in 9 mL of 37 °C X-VIVO 15 serum-free cell culture medium (Lonza, Basel, Switzerland). The cells were pelleted via centrifugation at 200× *g* for 10 min, the supernatant was removed, and the pellet was resuspended in 10 mL of 37 °C culture medium before determining live cell concentration with Trypan Blue (Gibco Laboratories, Gaithersburg, MD, USA) and manual hemocytometer counts. The cell concentration was adjusted to 166,667 cells/mL with additional 37 °C medium to achieve desired seeding number (50,000 cells/well) before addition of Dynabeads Human T-Activator CD3/CD28 activation beads (Gibco Laboratories) in a 1:1 (bead:cell) ratio. The solution was then plated in 300 µL volumes on two round-bottom polypropylene 96-well plates from Corning Life Sciences (Corning, NY, USA) and incubated at 37 °C for three days.

### 4.2. Growth Plate

After the three day post-thaw acclimation, recombinant human IL-2, IL-7, and IL-15, sourced from Miltenyi Biotec (Bergisch Gladbach, Germany) and diluted in sterile-filtered water, were pipetted into individual wells at the 16 experimental concentrations below to give quadruplicate wells per cytokine condition. The key index, along with the actual concentration of each cytokine is listed in Table 2. One well of each condition was sampled on days 3, 5, and 6 via Trypan Blue exclusion and hemocytometer count to determine cell concentration, and every well was sampled on days 4 and 7.

Growth rates were calculated between days three and seven except for the third data set, where day six was chosen as the endpoint. In that experiment, many of the conditions reached prohibitively dense populations (approaching or exceeding 10^6^ cells/mL) by day six, and consistently declined afterward to day seven, suggesting depletion of medium nutrients or other limitations of overly-confluent cultures. The first three days of the culture were not included in calculations as growth is stagnated during this period due to media acclimation and post-activation anergy. Simple exponential growth was assumed and growth rates were calculated as:

(1) $$G\;=\;\frac{\ln\left(\frac{c_{2}}{c_{1}}\right)}{\left(t_{2}-t_{1}\right)}$$

where $c_{1}$ and $c_{2}$ are live cell concentrations at times $t_{1}$ and $t_{2}$ respectively. The growth rates of each condition were then normalized to the average growth rate of that respective data set, as there were inter-experiment differences in maximum cell concentrations (please refer to Section 3.2).

### 4.3. Transcriptomics Analysis

Eighteen wells each of conditions 000, 200, 020, and 002 (as shown in Table 2) were replicated on the second 96-well plate for RNA extraction at three time points (days 4, 5 and 7). At each time point 6 wells for each of these conditions were pooled and the cells lysed with a RNeasy Plus Mini kit (Qiagen, Hilden, Germany). The lysates were frozen at −70 °C until RNA isolation could be performed. Sequencing of the isolated total RNA was performed by the Center for Medical Genomics (Indianapolis, IN, USA). The following protocol was used for RNA seq analysis.

After obtaining the FASTQ data, it had to be processed and examined. In order to process the FASTQ data, alignment software was used. STAR software was the chosen software. STAR stands for Spliced Transcripts Alignment to a Reference, which is a novel software RNA-seq alignment algorithm that uses “sequential maximum mappable seed search in uncompressed suffix arrays followed by seed clustering and stitching procedure” [36]. Subsequently, NGSUTils software suite was used. NGSUTils is a software tool suite for examining data from RNA Sequencing experiments, such as FASTQ files, which is what was used as the initial reads from the RNA Sequencing data from this experiment [37]. *featureCounts* is a software first introduced in *Bioinformatics.* The software is a read summarization program used for counting reads produced from RNA Sequencing tests. The highlight from *featureCounts* is that it utilizes chromosome hashing and feature blocking techniques. The software works with single and paired-end reads [38]. Finally, edgeR was used to analyze the gene expression data and perform several analytical functions to examine the count data [39]. On the basis of the expression profiles returned by edgeR, principal component analysis (PCA), one of the most popular approaches to project high-dimensional data into a two-dimensional space, was used to determine the outliner genes shown in Figure 4A,C,E. In particular, the PCA approach published in our previous work [40,41,42] was implemented in this work.

### 4.4. Fuzzy Modeling

On the basis of the growth data, a Fuzzy model was developed to predict the growth rate of activated CD4^+^ T cells stimulated by IL-2, IL-7 and IL-15. In the fuzzy model, the value of each of the inputs (i.e., concentrations of IL-2, IL-7, and IL-15) and the output (i.e., the growth rate of activated CD4+ T cells) was categorized into Low or High label (to reduce the number of model parameters). The growth data was then fed into the Fuzzy model so that an Adaptive Neuro Fuzzy Inference System [32,33,34,43] was to build the linguistic rules to link the inputs and output. One example linguistic rule is “*If IL-2 concentration is high, IL-7 concentration is low, and IL-15 concentration is low, the growth rate is high*”. A membership function (e.g., Gaussian function) was assigned for each linguistic label (e.g., Low) of each variable (i.e., three inputs and output) to quantify the probability of that variable falling into each category. Then Fuzzy model then infers the value of the output on the basis of the variables’ membership functions and linguistic rules. Figure 6 shows an illustrative diagram of the Fuzzy model.

The Fuzzy modeling approach was selected over other models like simpler linear regression models due to the following reasons. Firstly, the fuzzy model can return linguistic rules that can be understood easily. The inference system in the fuzzy approach is the significant similarity to human logic and decision-making, as the fuzzy approach can take these inconsistencies into account and help create inferences where a human mind may fall short. In addition, machine learning algorithms for Fuzzy models have been well studied so that the developed model has good prediction capability. As compared to a Mamdani Fuzzy Inference System that is adaptive, linear regression does not take into account the non-linearities, and is not easily adjustable with new information.

From the growth data, a Mamdani fuzzy model was created using the MATLAB command *tunefis*, whereby the Fuzzy Interference System was developed using the bounds of the experiment for each of the cytokines’ concentrations. In this instance, the bounds for the cytokine concentrations were 40 ng/mL, 100 ng/mL and 100 ng/mL for IL-2, IL-7 and IL-15, respectively. After the Fuzzy Interference System (FIS) was created and the membership functions were established, the parameters in variables’ membership functions were tuned to minimizes the root-mean squared error and get a global minimum solution using the particle swarm algorithm [44,45,46].

After the rules were established and membership functions were estimated, the Fuzzy model was evaluated by testing the growth data against the model for eight new conditions (inputs). After the Fuzzy model was validated, in order to test all possible combinations of cytokines between the aforementioned bounds set forth, a 3-D surface scatter heat map was developed to predict the conditions that would produce the highest growth rate of the T cells from the Fuzzy model. In Figure 3, the bright yellow color represents the high growth conditions while the blue represents the low growth conditions.

## 5. Conclusions

To conclude, we have investigated the growth of activated CD4^+^ T cells via the influence of three key cytokines: IL-2, IL-7 and IL-15. Through a design of experiments with different cytokine concentrations, we identified several key conditions to optimize the growth of the activated CD4^+^ T cells. The most favorable conditions for T cell growth study, and from the predictive model align to include 10 ng/mL for IL-2 and 36 ng/mL for IL-15, followed by 36 ng/mL for IL-7 and 36 ng/mL for IL-15, followed by 10 ng/mL of IL-2 and 36 ng/mL for IL-15. These conditions led to the highest growth rate of activated CD4^+^ T cells in our study with statistical significance. The Fuzzy Inference System successfully analyzed the growth of the cells under the influence of the cytokines, and to predicts moderate concentrations of synergetic cytokine cocktails to optimize the growth of the cells. To explore and support the result, we also utilized RNA Sequencing analysis to take several gene expression snapshots to understand the impact of each cytokine on the genes up- and down-regulated from the cells during the experiment. We found genes relating to different signaling pathways associated with T cell growth, as well as specific signaling pathways that each cytokine influenced in the T cells using principal component analysis, GeneMANIA and MetaCore. These signaling pathway include TCR, JAK/STAT, MAPK, AKT and PI3K-AKT signaling. In particular, IL-2 regulated genes involved in cell and leukocyte chemotaxis, chemokine receptor activity, cellular defense response and cell motility. IL-7 regulated the genes associated with chloride transport, cell chemotaxis, chloride transmembrane activity, and cytokine receptor activity, and IL-15 regulated genes for cell growth, zinc ion responses, cellular response to metal ions, and regulation of calcium ion transport into the cell. Lastly, growth data analysis indicated a statistically significant interaction between IL-2 and IL-7, reproduced in all data sets and lays the groundwork for expositing the combination of these two cytokines and how they act synergistically together for the CD4^+^ T cell phenotype. We recommend a system for clinicians to utilize to accelerate the growth of initially naïve CD4^+^ T cells and lay the groundwork for an approach to identifying optimized growth potential in other types of human leukocytes.

## Figures and Tables

**Table 1:** Pairwise *t*-tests for the growth rates between the 16 conditions, using the growth rate data shown in Figure 1. *p*-values are shown at the intersection of the row and column for each condition pair. The table was generated using R studio’s “pairwise.t.test” command, and the assumptions of normality and homogeneity of variances were verified using the Shapiro-Wilk and Bartlett tests, respectively. Statistical differences between means were identified for α < 0.05.

	000	001	002	010	011	020	022	100	101	110	111	200	202	220	222
001	0.282	-	-	-	-	-	-	-	-	-	-	-	-	-	-
002	0.293	0.890	-	-	-	-	-	-	-	-	-	-	-	-	-
010	0.214	0.874	0.755	-	-	-	-	-	-	-	-	-	-	-	-
011	0.029	0.340	0.225	0.433	-	-	-	-	-	-	-	-	-	-	-
020	0.060	0.435	0.323	0.533	0.917	-	-	-	-	-	-	-	-	-	-
022	0.126	0.662	0.538	0.781	0.629	0.728	-	-	-	-	-	-	-	-	-
100	0.164	0.763	0.640	0.887	0.528	0.629	0.892	-	-	-	-	-	-	-	-
101	0.043	0.428	0.300	0.534	0.853	0.951	0.750	0.640	-	-	-	-	-	-	-
110	0.020	0.270	0.169	0.350	0.864	0.797	0.525	0.434	0.722	-	-	-	-	-	-
111	0.124	0.657	0.533	0.775	0.635	0.734	0.994	0.886	0.756	0.531	-	-	-	-	-
200	0.035	0.376	0.255	0.474	0.937	0.974	0.679	0.575	0.916	0.802	0.685	-	-	-	-
202	0.075	0.493	0.376	0.597	0.835	0.923	0.801	0.699	0.965	0.717	0.807	0.890	-	-	-
220	0.584	0.551	0.608	0.443	0.090	0.154	0.287	0.358	0.128	0.064	0.283	0.105	0.184	-	-
222	0.240	0.925	0.809	0.948	0.393	0.492	0.732	0.836	0.489	0.316	0.726	0.432	0.553	0.485	-
no cyt	0.878	0.227	0.231	0.170	0.021	0.046	0.098	0.129	0.031	0.014	0.096	0.025	0.057	0.485	0.192

**Table 2:** The keys and concentrations of IL-2, IL-7 and IL-15 used in the experiments. Experimental concentrations were chosen from literature precedent and refined from initial growth studies and modeling results.

Key	IL-2 (ng/mL)	IL-7 (ng/mL)	IL-15 (ng/mL)
PC	0	0	0
0,0,0	1	6	6
1,0,0	10	6	6
2,0,0	15	6	6
0,1,0	1	36	6
0,2,0	1	80	6
0,0,1	1	6	36
0,0,2	1	6	80
1,1,0	10	36	6
1,0,1	10	6	36
0,1,1	1	36	36
2,2,0	15	80	6
2,0,2	15	6	80
0,2,2	1	80	80
1,1,1	10	36	36
2,2,2	15	80	80

**Table 3:** Statistical analysis of interaction of IL-2, IL-7, and IL-15 in influencing activated CD4^+^ T-cell growth rates by factorial analysis of variance (ANOVA). The analysis was conducted with R Studio’s “aov” command, and normality of residuals and homogeneity of variances were verified using the Shapiro and Bartlett tests, respectively. Pr (>F) of less than 0.05 was used to test for significance of interaction between interleukins. Significant interaction between IL-2 and IL-7 indicates that the effect of IL-2 is not consistent across all levels of IL-7, and the converse, suggesting an interactive effect of both present.

	Df	Sum Sq	Mean Sq	F Value	Pr (>F)
IL-2	1	0.049	0.0486	0.533	0.4688
IL-7	1	0.030	0.0303	0.333	0.5667
IL-15	1	0.012	0.0124	0.136	0.7137
**IL-2:IL-7**	**1**	**0.618**	**0.6180**	**6.785**	**0.0122**
IL-2:IL-15	1	0.231	0.2305	2.531	0.1182
IL-7:IL-15	1	0.003	0.0030	0.033	0.8560
**IL-2:IL-7:IL-15**	**1**	**0.412**	**0.4122**	**4.525**	**0.0386**
Residuals	48	4.372	0.0911		

**Table 4:** The functions of metabolic genes involved in positive and negative proliferation that have large change in their expression levels. Data for up- and down-regulated genes were obtained from RNA sequencing of samples supplemented at the no cytokine, 000, 200, 020, and 002 conditions. Genes were identified using the program Metacore and referenced with several databases, including the Biological Magnetic Resonance Data Bank and the Mammalian Metabolic Enzyme Database.

Genes for Positive Proliferation	Functions of Genes	Genes for Negative Proliferation	Functions of Genes
*PFKFB1*	Carbohydrate metabolism	*KIFC3*	Metabolism of Proteins
*APOE*	Lipid metabolism	*MGLL*	Lipid metabolism
*PLA2G6*	Lipid metabolism	*CHST1*	Amino Acid Metabolism
*TPTE2*	Lipid metabolism	*CDO1*	Amino Acid Metabolism
*OLAH*	Citrate & Glyoxylate cycle	*MRM1*	RNA Metabolism
*ACHE*	Lipid Metabolism	*AICDA*	Nucleotide Metabolism
*BAAT*	Steroid Metabolism	*SMPD3*	Lipid Metabolism
*LDHC*	Amino Acid Metabolism	*VEGFC*	Growth Factor
*RDH5*	Lipid metabolism	*PTHLH*	Hormone
*ACAD11*	Enzyme	*SFRP2*	Wnt Signaling
*PRKAA2*	Lipid Metabolism		
*CES3*	Lipid Metabolism		
*DMGDH*	Lipid Metabolism		
*EGR4*	Growth Factor		
*KLK2*	Protein metabolism		
*TSHR*	Thyroid cell metabolism		
*TGFB2*	Growth Factor		

**Table 5:** The outlier genes shown in Figure 4 that are involved in TCR signaling. The data were generated through RNA sequencing of samples for the no cytokine, 000, 200, 020, and 002 conditions, and the listed genes were identified from that set through principal component analysis.

Cytokine Stimulation	Outlier Gene from PCA	CD4+ Naïve TCR Gene
IL-2	CXCL8	PIK3CA
IL-2	CXCL8	SHC1
IL-2	CXCL8	SLA2
IL-2	CXCL8	SOS1
IL-2	VEGFC	PIK3CA
IL-2	VEGFC	SHC1
IL-2	VEGFC	SOS1
IL-7	CACNA1E	RASGRP2
IL-7	CXCL8	PIK3CA
IL-7	CXCL8	SHC1
IL-7	DPYSL5	CRMP1
IL-7	CXCL8	SLA2
IL-7	CXCL8	SOS1
IL-7	ITGB8	FLNA

## Supplementary Materials

Supplementary Materials can be found at https://www.mdpi.com/1422-0067/21/21/7814/s1.

## References

[1] Das K.R., Vernau L., Grupp S.A., Barrett D.M. (2019). Naive T-cell Deficits at Diagnosis and after Chemotherapy Impair Cell Therapy Potential in Pediatric Cancers. Cancer Discov..

[2] Read K.A., Powell M.D., McDonald P.W., Oestreich K.J. (2016). IL-2, IL-7 and IL-15: Multistage regulators of CD4+ T helper cell differentiation. Exp. Hematol..

[3] Geginat J., Sallusto F., Lanzavecchia A. (2001). Cytokine-driven Proliferation and Differentiation of Human Naive, Central Memory, and Effector Memory CD4+ T Cells. J. Exp. Med..

[4] Jaleco S., Swainson L., Dardalhon V., Burjanadze M., Kinet S., Taylor N. (2003). Homeostasis of Naive and Memory CD4+ T Cells: IL-2 and IL-7 Differentially Regulate the Balance Between Proliferation and Fas-Mediated Apoptosis. J. Immunol..

[5] Alves L.N., Hooibrink B., Arosa F.A., van Lier R.A.W. (2003). IL-15 induces antigen-independent expansion and differentiation of human naive CD8+ T cells in vitro. Blood.

[6] Unutmaz D., Pileri P., Abrignani S. (1994). Antigen-Independent Activation of Naive and Memory Resting T Cells by a Cytokine Combination. J. Exp. Med..

[7] Kanegane H., Tosato G. (1996). Activation of Naive and Memory T Cells by Interleukin-15. Blood.

[8] Golubovskaya V., Wu L. (2016). Different Subsets of T Cells, Memory, Effector Functions, and CAR-T Immunotherapy. Cancers.

[9] Sommermeyer D., Hudacek M., Kosasih P.L., Gogishvili T., Maloney D.G., Turtle C.J., Riddell S.R. (2016). Chimeric antigen receptor-modified T cells derived from defined CD8^+^ and CD4^+^ subsets confer superior antitumor reactivity in vivo. Leukemia.

[10] Wang D., Aguilar B., Starr R., Alizadeh D., Brito A., Sarkissian A., Ostberg J.R., Forman S.J., Brown C.E. (2018). Glioblastoma-targeted CD4+ CAR T cells mediate superior antitumor activity. JCI Insight.

[11] Litvinova L.S., Sokhonevich N.A., Gutsol A.A., Kofanova K.A. (2013). The Influence of Immunoregulatory Cytokines IL-2, IL-7, and IL-15 upon Activation, Proliferation, and Apoptosis of Immune Memory T-cells in vitro. Cell Tissue Biol..

[12] Silva S.L., Albuquerque A.S., Matoso P., Charmeteau-de-Muylder B., Cheynier R., Ligeiro D., Abecasis M., Anjos R., Barata J.T., Victorino R.M. (2017). IL-7-Induced Proliferation of Human Naive CD4 T-Cells Relies on Continued Thymic Activity. Front. Immunol..

[13] Dooms H., Abbas A.K. (2006). Control of CD4+ T-cell memory by cytokines and costimulators. Immunol. Rev..

[14] Kinter A.L., Godbout E.J., McNally J.P., Sereti I., Roby G.A., O’Shea M.A., Fauci A.S. (2008). The Common Gamma-Chain Cytokines IL-2, IL-7, IL-15, and IL-21 Induce the Expression of Programmed Death-1 and Its Ligands. J. Immunol..

[15] Fisher R.A. (1921). 014: On the ‘Probable Error’ of a Coefficient of a Correlation Deduced from a Small Sample. Metron.

[16] Mamdani E.H., Assilian S. (1975). An Experiment in Linguistic Synthesis with a Fuzzy Logic Controller. Int. J. Man-Mach. Stud..

[17] Wold S., Esbensen K., Geladi P. (1987). Principal Component Analysis. Chemom. Intell. Lab. Syst..

[18] Warde-Farley D., Donaldson S.L., Comes O., Zuberi K., Badrawi R., Chao P., Franz M., Grouios C., Kazi F., Lopes C.T. (2010). The GeneMANIA Prediction server: Biological network integration for gene prioritization and predicting gene function. Nucleic Acids Res..

[19] Dubovenko A., Nikolsky Y., Rakhmatulin E., Nikolskaya T. (2017). Functional Analysis of OMICs Data and Small Molecule Compounds in an Integrated Knowledge-Based Platform. Biological Networks and Pathway Analysis.

[20] TCR Signaling in Naive CD4 T Cells. NDEx. http://www.ndexbio.org/#/network/0c2862fa-6196-11e5-8ac5-06603eb7f303.

[21] Tokunaga R., Zhang W., Naseem M., Puccini A., Berger M.D., Soni S., McSkane M., Baba H., Lenz H.J. (2018). CXCL9. CXCL10, CXCL11/CXCR3 axis for immune activation—A target for novel cancer therapy. Cancer Treat. Rev..

[22] Chien M., Ku C., Johansson G., Chen M., Hsiao M., Su J., Inoue H., Hua K., Wei L., Kuo M. (2009). Vascular Endothelial Growth Factor-C (VEGF-C) promotes angiogenesis by induction of COX-2 in leukemic cells via the VEGF-R3/JNK/AP-1 pathway. Carcinogenesis.

[23] Tacconi C., Ungaro F., Correale C., Arena V., Massimino L., Detmar M., Spinelli A., Carvello M., Mazzone M., Oliveira A.I. (2019). Activation of the VEGFC/VEGFR3 Pathway Induces Tumor Immune Escape in Colorectal Cancer. Cancer Res..

[24] Rochman Y., Spolski R., Leonard W.J. (2009). New Insights into the regulation of T cells by γc Family Cytokines. Nat. Rev. Immunol..

[25] Krebs R., Tikkanen J.M., Ropponen J.O., Jeltschm M., Jokinen J.J., Yla-Herttuala S., Nykanen A.I., Lemstrom K.B. (2012). Critical Role of VEGF-C/VEGFR-3 Signaling in Innate and Adaptive Immune Responses in Experimental Obliterative Bronchiolitis. Am. J. Pathol..

[26] Mason E.F., Rathmell J.C. (2011). Cell metabolism: An essential link between growth and apoptosis. Biochim. Biophys. Acta.

[27] Rauniyar K., Jha S.J., Jeltsch M. (2018). Biology of Vascular Endothelial Growth Factor C in the Morphogenesis of Lymphatic Vessels. Front. Bioeng. Biotechnol..

[28] Luther S.A., Cyster J.G. (2001). Chemokines as regulators of T cell differentiation. Nat. Immunol..

[29] Zhan Y., Carrington E.M., Zhang Y., Heinzel S., Lew A.M. (2017). Life and Death of Activated T Cells: How Are They Different From Naïve T Cells?. Front. Immunol..

[30] Lee D.W., Gardner R., Porter D.L., Louis C.U., Ahmed N., Jensen M., Grupp S.A., Mackall C.L. (2014). Current concepts in the diagnosis and management of cytokine release syndrome. Blood.

[31] Suntharalingam G., Perry M.R., Ward S., Brett S.J., Castello-Cortes A., Brunner M.D., Panoskaltsis N. (2006). Cytokine storm in a phase 1 trial of the anti-CD28 monoclonal antibody TGN1412. N. Engl. J. Med..

[32] Hay K.A., Hanafi L.A., Li D., Gust J., Liles W.C., Wurfel M.M., López J.A., Chen J., Chung D., Harju-Baker S. (2017). Kinetics and biomarkers of severe cytokine release syndrome after CD19 chimericantigen receptor-modified T-cell therapy. Blood.

[33] Conti P., Ronconi G., Caraffa A., Gallenga C.E., Ross R., Frydas I., Kritas S.K. (2020). Induction of Pro-inflammatory Cytokines (IL-1 and IL-6) and Lung Inflammation by Coronavirus-19 (COVI-19 or SARS-CoV-2): Anti-inflammation Strategies. J. Biol. Regul. Homeost. Agents.

[34] Caraffa A., Gallenga C.E., Kritas S.K., Ronconi G., Di Emidio P., Conti P. (2019). CAR-T Cell Therapy Causes Inflammation by IL-1 which Activates Inflammatory Cytokine Mast Cells: Anti-inflammatory Role of IL-37. J. Biol. Regul. Homeost. Agents.

[35] Kritas S.K., Ronconi G., Caraffa A., Gallenga C.E., Ross R., Conti P. (2020). Mast Cells Contribute to Coronavirus-induced Inflammation: New Anti-inflammatory Strategy. J. Biol. Regul. Homeost. Agents.

[36] Dobin A., Davis C.A., Schlesinger F., Drenkow J., Zaleski C., Jha S., Batut P., Chaisson M., Gingeras T.R. (2012). STAR: Ultrafast Universal Rna-Seq Aligner. Bioinformatics.

[37] Breese M.R., Liu Y. (2013). NGSUtils: A Software Suite for Analyzing and Manipulating Next-Generation Sequencing Datasets. Bioinformatics.

[38] Liao Y., Smyth G.K., Shi W. (2013). FeatureCounts: An Efficient General Purpose Program for Assigning Sequence Reads to Genomic Features. Bioinformatics.

[39] Robinson M.D., McCarthy D.J., Smyth G.K. (2010). edgeR: A Bioconductor package for differential expression analysis of digital gene expression data. Bioinformatics.

[40] Hua M., Huang W., Chen A., Rehmet M., Jin C., Huang Z. (2020). Comparison of Antimicrobial Resistance Detected in Environmental and Clinical Isolates from Historical Data for the US. BioMed Res. Int..

[41] Yang K., Wang A., Fu M., Wang A., Chen K., Jia Q., Huang Z. (2020). Investigation of Incidents and Trends of Antimicrobial Resistance in Foodborne Pathogens in Eight Countries from Historical Sample Data. Int. J. Environ. Res. Public Health.

[42] Shing J., Jang R. (1993). ANFIS: Adaptive-Network-Based Fuzzy Inference System. IEEE Trans. Syst. Man Cybern..

[43] Teachey D.T., Lacey S.F., Shaw P.A., Melenhorst J.J., Maude S.L., Frey N., Pequignot E., Gonzalez V.E., Chen F., Finklestein J. (2016). Identification of Predictive Biomarkers for Cytokine Release Syndrome after Chimeric Antigen Receptor T-cell Therapy for Acute Lymphoblastic Leukemia. Cancer Discov..

[44] Ghanbari A., Ghaderi S.F., Azadeh M.A. Adaptive Neuro-Fuzzy Inference System vs. Regression Based Approaches for Annual Electricity Load Forecasting. Proceedings of the 2nd International Conference on Computer and Automation Engineering.

[45] Esmaeli M., Osanloo M., Rashidinejad F., Bazzazi A.A., Taji F. (2014). Multiple regression, ANN and ANFIS models for prediction of backbreak in the open pit blasting. Eng. Comput..

[46] Mokarram M., Amin H., Khosravi M.R. (2019). Using Adaptive Neuro-Fuzzy Inference System and Multiple Linear Regression to Estimate Orange Taste. Food Sci. Nutr..

