# Supplementary material for: Investigation of the Impact from IL-2, IL-7, and IL-15 on the Growth and Signaling of Activated CD4+ T Cells
Source: Int J Mol Sci. 2020 Oct 22;21(21):7814. doi: 10.3390/ijms21217814 (PMC7659484; doi:10.3390/ijms21217814)
Supplement: Supplementary file 1 [file ijms-21-07814-s001.pdf]

Supplementary Materials: Supplementary materials can be found at www.mdpi.com/1422-0067/21/21/7814/s1.

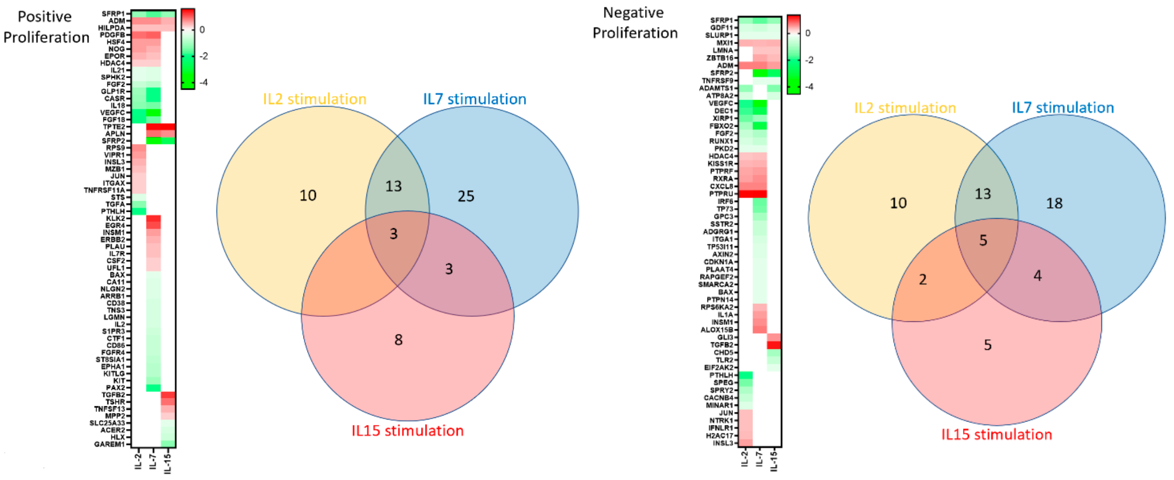

(A) (B)

Figure 1. Significantly changed transcripts associated with proliferation related ontology vs. the control condition: (A) positive proliferation and (B) negative proliferation.

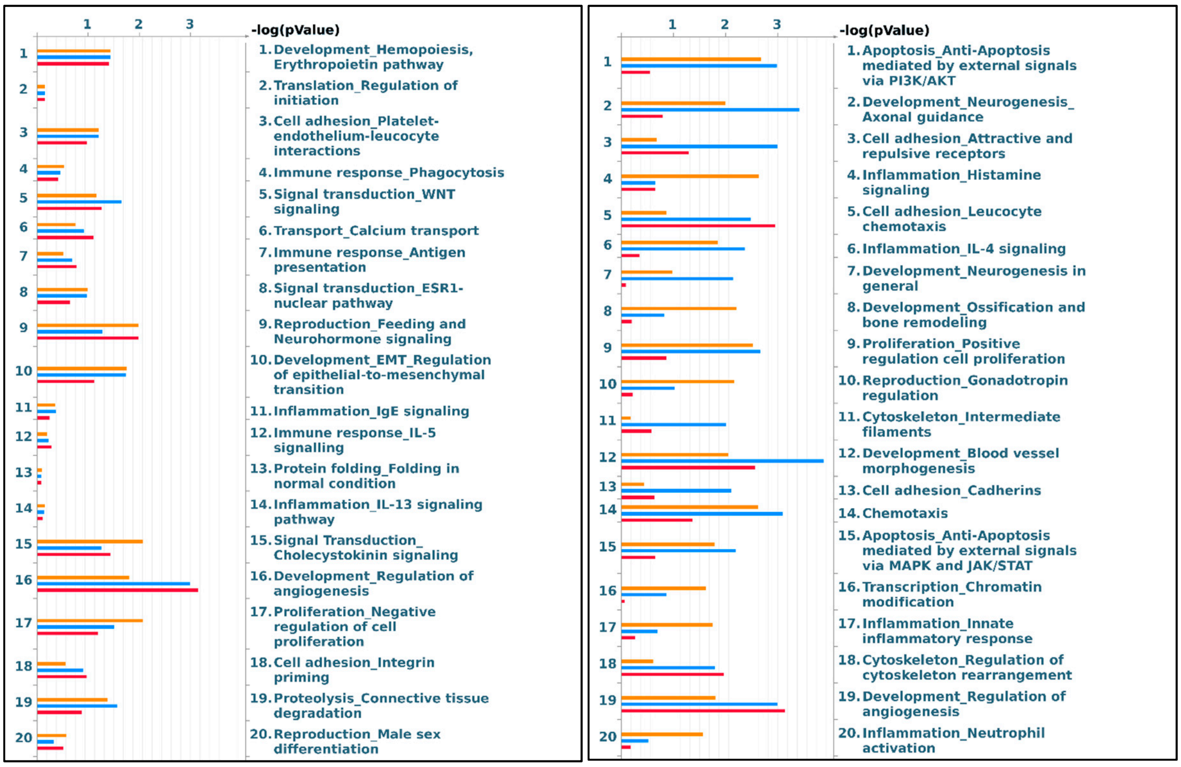

(A) (B)

Figure 2. The most statistically affected genetic networks either shared (Supplementary Figure S2A) or uniquely changed (Supplementary Figure S2B) between conditions: orange for IL2, blue for IL7, and red for IL15.
